# Anterior *Hox* Genes Interact with Components of the Neural Crest Specification Network to Induce Neural Crest Fates

**DOI:** 10.1002/stem.630

**Published:** 2011-03-23

**Authors:** Mina Gouti, James Briscoe, Anthony Gavalas

**Affiliations:** a Developmental Biology Laboratory, Biomedical Research Foundation of the Academy of Athens (BRFAA)Athens, Greece; b Division of Developmental Neurobiology, MRC National Institute for Medical Research (NIMR)The Ridgeway, Mill Hill, London, United Kingdom

**Keywords:** *Hox* genes, Neural crest specification, Epithelial mesenchymal transition, Snail genes, Bmp and Notch signaling

## Abstract

*Hox* genes play a central role in neural crest (NC) patterning particularly in the cranial region of the body. Despite evidence that simultaneous loss of Hoxa1 and Hoxb1 function resulted in NC specification defects, the role of *Hox* genes in NC specification has remained unclear due to extended genetic redundancy among *Hox* genes. To circumvent this problem, we expressed anterior *Hox* genes in the trunk neural tube of the developing chick embryo. This demonstrated that anterior *Hox* genes play a central role in NC cell specification by rapidly inducing the key transcription factors Snail2 and Msx1/2 and a neural progenitor to NC cell fate switch characterized by cell adhesion changes and an epithelial-to-mesenchymal transition (EMT). Cells delaminated from dorsal and medial neural tube levels and generated ectopic neurons, glia progenitors, and melanocytes. The mobilization of the NC genetic cascade was dependent upon bone morphogenetic protein signaling and optimal levels of Notch signaling. Therefore, anterior Hox patterning genes participate in NC specification and EMT by interacting with NC-inducing signaling pathways and regulating the expression of key genes involved in these processes. Stem Cells 2011;29:858–870

## INTRODUCTION

Neural crest (NC) cells originate at the interface between the neural plate and the non-neural ectoderm and are characterized by their multipotency and migratory capacity. They migrate extensively into the developing embryo differentiating into a wide array of cell types including neurons and glia of the peripheral nervous system, pigment cells, and facial cartilage and bones in the cranial region of the body [1]. NC stem cells appear to be maintained in different tissues of the adult and can generate neurons, Schwann cells, myofibroblasts, chondrocytes, and melanocytes. Their wide developmental potential and regenerative capacity have stimulated interest in using them in stem cell-based therapies but their isolation, expansion, and generation remain challenging [2]. Elucidating the mechanisms underlying their generation could bring their use in regenerative medicine closer.

NC cells are specified in response to extrinsic signals and intrinsic factors. Bone morphogenetic protein (Bmp), Wnt, and Notch signaling have been implicated in NC cell induction through the activation of key transcription factors [3, 4]. Fibroblast growth factor (Fgf) and retinoic acid (RA) signaling have been implicated in NC induction concomitantly with inducing posterior fates in the neural plate [5] and their expression in paraxial mesoderm [6]. Targets of their action in NC induction have not been identified, but both Fgf and RA signaling regulate expression of *Hox* genes either directly or through Cdx transcription factors [7–10]. *Hox* genes are expressed at the time of NC induction [11] but extensive functional redundancy among members of this family might have masked their role in this process.

A battery of transcription factors such as Msx1/2, Gbx2, Pax3, and Pax7, are induced in the neural plate border in response to signaling from non-neural ectoderm and paraxial mesoderm and establish a region of competence for NC cell specification. In turn, a second set of transcription factors such as Snail, Sox9, Sox10, and FoxD3 [12] are induced and implement the genetic program defining NC cells. This includes an epithelial-to-mesenchymal transition (EMT) mediated by reorganization of the actin cytoskeleton, loss of epithelial polarity, and alterations in cell adhesion properties [13].

Despite similarities in the molecular mechanisms underlying NC specification in cranial and trunk regions there are important differences including expression patterns of key genes and the order of their activation during NC specification [14–16]. Several gain and loss-of-function experiments result in distinct phenotypes in the cranial and the trunk region [14, 17–19]. *Hox* patterning genes are expressed from early neurulation stages in the developing neuroepithelium as well as the emerging hindbrain and trunk NC and may account for these differences but extensive functional redundancy in NC specification may have masked their implication in this process. Loss-of-function of single or multiple *Hox* genes generally resulted in patterning defects but not NC specification or migration defects [20]. However, a rhombomere 4 (r4)-specific triple Hox loss-of-function mutation resulted in loss of the expression of all r4 NC-derived molecular markers and all the structures normally derived from r4 NC populating the second arch. Reciprocal grafts between mutant and wt embryos showed that these defects were cell autonomous to the mutant r4 implying a loss of NC cell specification [21, 22]. In a screen designed to identify potential Hoxb1 target genes and processes in embryonic stem cell-derived neural stem (NS) cells, we found that *Msx1/2* and *Snail1* as well as dorsal progenitor markers were upregulated in response to Hoxb1 expression [23]. These findings raised the possibility that anterior *Hox* genes may be directly involved in NC specification. Here, we investigated this issue and found that, when expressed in the neural tube of the trunk region of the developing chick embryo, anterior *Hox* genes can induce NC cell fates to variable extents. *Hoxb1* can impose a neural to NC cell fate switch accompanied by a reduction in proliferation and changes in cell adhesion that lead to EMT. Hoxb1^+^ cells delaminated from both dorsal and medial levels of the neural tube and generated ectopic neuronal cells, glia progenitors, and melanocytes. Hoxb1-induced EMT and NC cell fate switch were dependent upon Bmp signaling and optimal levels of Notch signaling. Hoxb1 itself potentiated Notch signaling and repressed *Hes5* to induce NC specification and EMT. *Hoxa2* had a similar capacity in inducing NC cell character but a weaker one in inducing EMT. Other anterior *Hox* genes, such as *Hoxa1* and *Hoxb2*, but not the posterior gene *Hoxb4*, also had the capacity to induce NC cell character. These findings suggest that patterning genes participate in NC specification by interacting with signaling pathways and regulating the expression of key genes involved in NC specification and EMT.

## MATERIALS AND METHODS

### Differentiation of Mouse Embryonic Stem Cells (ESC) and Immunofluorescence

Differentiation of mouse ESC and immunofluorescence were performed as described earlier [23]. SB216763 (S3442, Sigma, Athens, Greece, http://www.sigmaaldrich.com/greece.html) was added at a final concentration of 10 μM at day 4 of the neuroepithelial selection stage until the end of the second day of the expansion phase at which point cells were analyzed.

### Chick In Ovo Electroporation

Fertilized chick eggs were electroporated at Hamburger Hamilton (HH) [24] stage 10–11. Plasmid DNA concentration was 1.5–μg/μl. As a control, pCAGGS-IRES-NLS-GFP was included at 0.5 μg/μl. Electroporation was carried out using a BTX ECM830 electroporator delivering five 20 V pulses of 50 millisecond duration each. Analysis in the spinal cord was carried out at cervical and upper thoracic levels.

### Expression Vectors

The coding regions of mouse *Hoxb1*, *Hoxb2*, *Hoxa1*, *Hoxa2*, and *Hoxb4* cDNAs were inserted into the pCAGGS-IRES-NLS-GFP expression vector [25] upstream of the IRES. The coding regions of *Hoxb2*, *Hoxa1*, and *Hoxa2* were fused in-frame with the hemagluttinin (HA) tag. The cDNAs were verified by sequencing and expression was verified after electroporation by immunofluorescence with antibodies recognizing the HA tag, *Hoxb1*, or *Hoxb4* proteins. Wnt and Bmp signaling were blocked using the *N-terminal deleted transcription factor 4* (*ΔN-TCF4*) and *noggin* chick expression vectors, respectively [26, 27]. Notch signaling was activated using an Notch intracellular domain (NICD) chick expression vector [28]. These plasmids were used at a final concentration of 2 μg/μl.

### *N*-[*N*-(3,5-Difluorophenacetyl-L-alanyl)]-*S*-phenylglycine τ-Butyl Ester (DAPT) Treatment, Luciferase, and BrdU Incorporation Assays

Embryos were treated 6h postelectroporation (PE) with 25 μl of the γ-secretase inhibitor DAPT at a concentration of 50 μM and collected 24h PE. To assay Wnt, Bmp, and Notch signaling activity the TOPflash (Millipore, Athens, Greece, http://www.millipore.com/offices/cp3/gr), 2× BRE [29], and 12× RBP-J [30] luciferase reporter plasmids respectively, were electroporated in the presence of a renilla luciferase expression plasmid (PROMEGA, Southampton, UK, http://www.promega.com/uk) and embryos were harvested 12h PE for chemiluminescence analysis. For BrdU incorporation, chick embryos were labeled by addition of 25 μl of 10 mM BrdU 4h PE and collected 6h PE.

### Statistical Analysis

All quantitative data were expressed as mean ± SD and significance levels were calculated using the Student's *t* test. For the BrdU analysis, the data shown represent the percentage of GFP^+^ in the electroporated area that are labeled with BrdU and the percentage of cells labeled with BrdU on the nonelectroporated side. All results presented in this report were from experiments repeated in at least five embryos for each stage analyzed.

### In Situ Hybridization and Immunofluorescence

In situ hybridization probes and antibodies used are listed in supporting information Materials and Methods. Whole mount in situ hybridization was followed by cryosectioning and immunofluorescence using standard methods. Images were acquired using a Leica (Mannheim, Germany, http://www.leica-microsystems.com) TCS SP5 confocal microscope.

## RESULTS

### Hoxb1 Induces Key Features of NC Fates in Trunk Neural Tube

**Figure 1:**
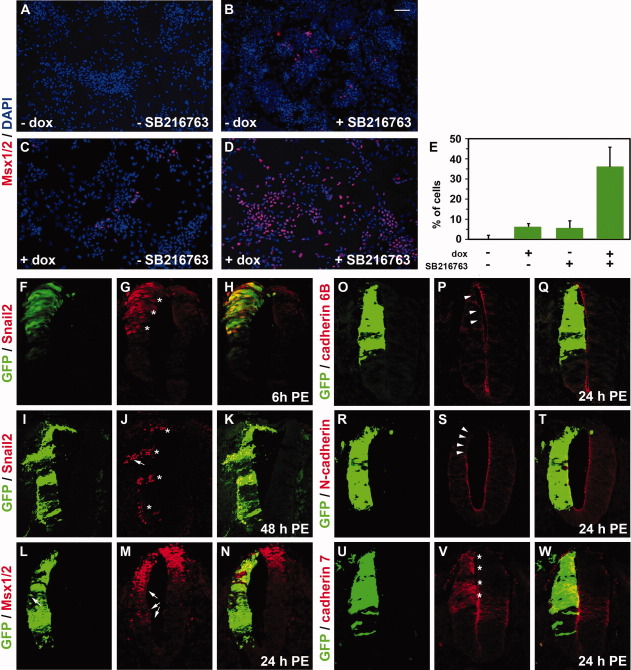
Expression of Hoxb1 induced Msx1/2 expression in embryonic stem-derived neural stem cells and induced sustained expression of Snail2 and Msx1/2 and altered cadherin expression in the chick trunk neural tube. Immunofluorescent detection of Msx1/2 (**A–D**) showed that its expression was slightly enhanced in Hoxb1^−^ cells (compare [**A**] with [**B**]) but dramatically upregulated in Hoxb1^+^ cells after induction of dorsal cell fates (compare [**C**] with [**D**]). Immunofluorescent detection of Snail2 (**F–K**), Msx1/2 (**L–N**) cadherin 6B (**O–Q**), N-cadherin (**R–T**), and cadherin 7 (**U–W**) on transverse sections of neural tubes 6h PE (**F–H**), 24h PE (**L–W**), and 48h PE (**I–K**) with Hoxb1. Hoxb1 upregulated Snail2 expression in a cell autonomous manner as early as 6h PE (asterisks in [**G**]) and maintained it at least until 48h PE (asterisks in [**J**]). Delaminating Snail2^+^ cells were seen at medial levels (arrow at [**J**]). Hoxb1 expression induced cell autonomously Msx1/2 upregulation 24h PE (arrows in [**M**]). It also repressed apical expression of cadherin 6B (arrowheads in [**P**]) and N-cadherin (arrowheads in [**S**]) at dorsal levels of the neural tube, whereas it induced strong upregulation of cadherin 7 (asterisks in [**V**]). Scale bar = 50 μm (**B**). Abbreviations: DAPI, 4′,6-diamidino-2-phenylindole; GFP, green fluorescent protein; PE, postelectroporation.

To understand the cellular processes and genes that *Hox* genes and *Hoxb1* in particular may control, we generated mouse ESC that allowed inducible expression of *Hoxb1*. Its timely induction in ESC-derived NS cells resulted in the specification of NS cells toward a hindbrain-specific identity. Molecular analyses suggested that Hoxb1^+^ embryonic stem (ES)–derived NS cells exhibited a preference for dorsal neural tube fates [23]. This could be attributed at least partly to an upregulation of endogenously generated dorsal signals such as Bmp4, Wnt1, and Wnt3A, which resulted in the upregulation of the NC genes *Snail1* and *Msx1* [23]. The homeodomain transcription factor Msx1 is a key inducer of NC cells and, together with Msx2, plays a crucial role in patterning and survival of cranial NC [31, 32]. To probe the possibility that Hoxb1 may synergize with NC-inducing signals to initiate the NC genetic program, we induced dorsal neural tube identity in Hoxb1^−^ and Hoxb1^+^ ES-derived NS cells by blocking glycogen synthase kinase 3β (GSK3β) activity using 10 μM of the specific antagonist SB216763 [33]. We found that although glycogen synthase kinase 3β (GSK3β) inhibition resulted in a small increase of *Msx1/2* expression in Hoxb1^−^ NS cells, *Msx1/2* expression in Hoxb1^+^ NS cells increased dramatically (Fig. 1A–1E). These data suggested that Hoxb1 participates in NC cell induction in vivo.

To further examine this in vivo, we introduced a *Hoxb1* expression vector into the developing trunk neural tube of chick embryos via in ovo electroporation at HH stage 10–11, a stage before NC cell delamination at that axial level. The *Snail* family genes encode zinc finger transcriptional repressors that are key regulators of EMT [34]. In the chick, Snail2 (a.k.a. as slug) is the functional homolog of the mouse Snail1 [14] and is essential for the induction of EMT [14, 35, 36]. We found that, after Hoxb1 electroporation, Snail2 was rapidly upregulated in a cell autonomous manner as early as 6h PE (Fig. 1F–1H) suggesting that this was a direct effect of Hoxb1 expression. Upregulation of Snail2 was maintained at 12h PE and 24h PE (data not shown), and was detected in migratory cells 48h PE (Fig. 1I–1K). It is worth noting that although NC specifiers, such as the group E Sox genes, can also induce Snail2 expression on their own, they do so transiently and without subsequent cell delamination [19, 37]. Consistent with NC cell fate induction, Msx1/2^+^ cells were detected ectopically located in more ventral positions of the neural tube at 24h PE (Fig. 1L–1N).

EMT involves loss of the apical basal polarity, a change in the expression of cell adhesion molecules, such as cadherins, as well as a radical reorganization of the cytoskeleton. This is incompatible with high proliferation rates, thus proliferation rates in NC cells are low and *Snail* genes induce EMT by blocking proliferation [38]. Thus, we examined whether electroporation of Hoxb1 and Snail2 induction were accompanied by cell adhesion changes and reduction of proliferation. Cadherin 6B is distributed apically in the neuroepithelium [39] and the induction of migratory NC cells relies upon the Snail2-dependent repression of its expression [40]. N-cadherin is also distributed apically in the neuroepithelium [41] and, similarly to cadherin 6B NC cell migration depends upon its downregulation [42]. Consistent with the initiation of an EMT after Hoxb1 electroporation, we found that both cadherin 6B and N-cadherin were downregulated in Hoxb1^+^ cells 24h PE (Fig. 1O–1Q, 1R–1T). On the other hand, cadherin-7 is expressed in migratory NC cells [39] and, consistent with induction of EMT, expression of Hoxb1 resulted in cadherin-7 upregulation 24h PE (Fig. 1U–1W).

The reduced size of the electroporated side of the neural tube (supporting information Fig. 1) could be due to cells undergoing EMT and leaving the neural tube, but it could also be due to a reduction in proliferation rates or cell death or both. To address this, dividing cells in electroporated embryos were labeled by addition of BrdU 4h PE and collected 6h PE. Hoxb1^+^ cells in the electroporated side showed a marked reduction in BrdU incorporation suggesting that Hoxb1 induced cell cycle withdrawal (supporting information Fig. 1). To investigate whether excessive cell death was also induced, we performed cleaved caspase-3 immunofluorescence assays at 24h PE but found similarly minimal staining on both Hoxb1 electroporated and nonelectroporated sides (data not shown).

These results argue that expression of Hoxb1 in the trunk neural tube induces key features of the NC genetic cascade including the swift and sustained expression of Snail2, induction of Msx1/2 expression, alteration of the cadherin expression profile, and a reduction in proliferation rates.

### Anterior Hox Transcription Factors Can Induce NC Cell Character with Variable Efficiencies

**Figure 2:**
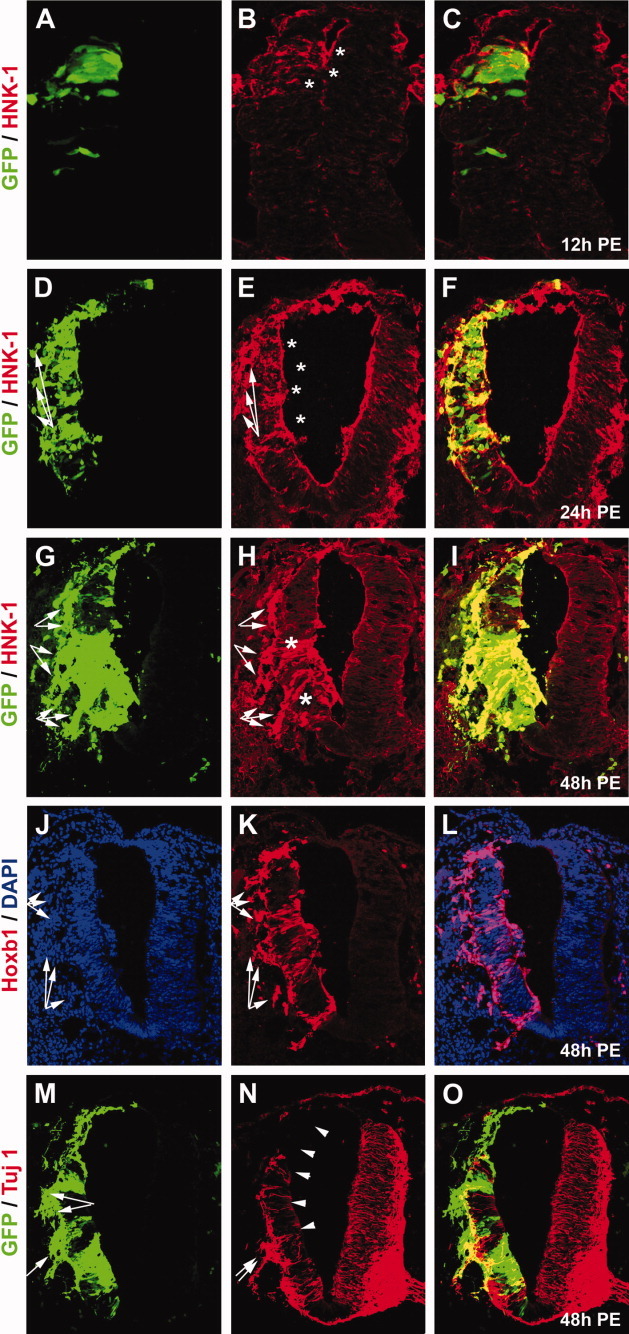
Expression of Hoxb1 unregulated HNK-1 and reduced neuronal differentiation. Immunohistochemical detection of HNK-1 (**A–I**), Hoxb1 (**J–L**), and Tuj1 (**M–O**) was performed on transverse sections of neural tubes 12h PE (**A–C**), 24h PE (**D–F**), and 48h PE (**G–O**) with Hoxb1. Hoxb1 upregulated HNK-1 ([**A–I**], asterisks in [**B, E, H**]) in a cell autonomous manner. Conversely, it strongly repressed neuronal differentiation as seen by Tuj1 immunofluorescence at 48h PE ([**M–O**], arrowheads in [**N**]). Electroporated cells delaminating ectopically could be seen as early as 24h PE (arrows in [**D, E**]) and then migrating away from the neural tube at 48h PE (arrows in [**G, H, J, K, M, N**]). Abbreviations: DAPI, 4′,6-diamidino-2-phenylindole; GFP, green fluorescent protein; HNK-1, human natural killer-1; PE, postelectroporation.

To determine whether other anterior *Hox* patterning genes are also able to induce NC cell specification, we introduced several anterior Hox expression vectors into the developing trunk neural tube of chick embryos via in ovo electroporation at HH stage 10-11. NC cell fate was examined using the premigratory and migratory avian NC cell marker human natural killer-1 (HNK-1), a surface epitope that has been used widely to identify NC cells [43]. We found that Hoxb1 and Hoxa2 strongly induced HNK-1 24h PE in a cell autonomous manner within the neural tube and disrupted the morphology of the neuroepithelium (Fig. 2D–2F, 7). Compared with Hoxb1, Hoxa1, and Hoxb2 had a relatively weaker capacity to induce HNK-1 (supporting information Fig. 1), whereas the more posterior Hoxb4 was not able to upregulate HNK-1 expression (supporting information Fig. 2). Ectopic HNK-1^+^ cells were present as early as 12h PE of Hoxb1 (Fig. 2A–2C) and appeared to be delaminating from the basal surface of the neural tube at 24h PE primarily in dorsal but also medial and ventral positions (Fig. 2D–2I). By 48h PE electroporated cells were observed migrating into the periphery (Fig. 2G–2L). The induction of NC markers correlated with reduced neuronal differentiation, as judged by β-III-tubulin expression (Fig. 2M–2O), suggesting a spinal cord to NC cell fate switch.

The differential effects of the tested *Hox* genes regarding HNK-1 induction suggested that effects are specific to distinct *Hox* genes rather than a consequence of general elevation of Hox protein levels. To further test this, we electroporated Hoxb1 in the Hox-free midbrain territory at HH stage 10-11 and assayed for upregulation of HNK-1, Snail2, Msx1/2, and β-III-tubulin. Consistent with the effects observed in the trunk, Hoxb1 expression in the midbrain resulted in upregulation of Snail2, Msx1/2, upregulation of HNK-1, and reduced neuronal differentiation indicated by reduced β-III-tubulin expression 24h PE (supporting information Fig. 3).

These findings suggested that anterior *Hox* genes might induce NC cell character with variable efficiencies. Among the genes tested, Hoxb1 showed the highest efficiency and to explore the mechanisms involved, we concentrated on analyzing its effects in NC induction.

### Hoxb1 Expression Induces Cell Fate Changes in the Trunk Neural Tube

**Figure 3:**
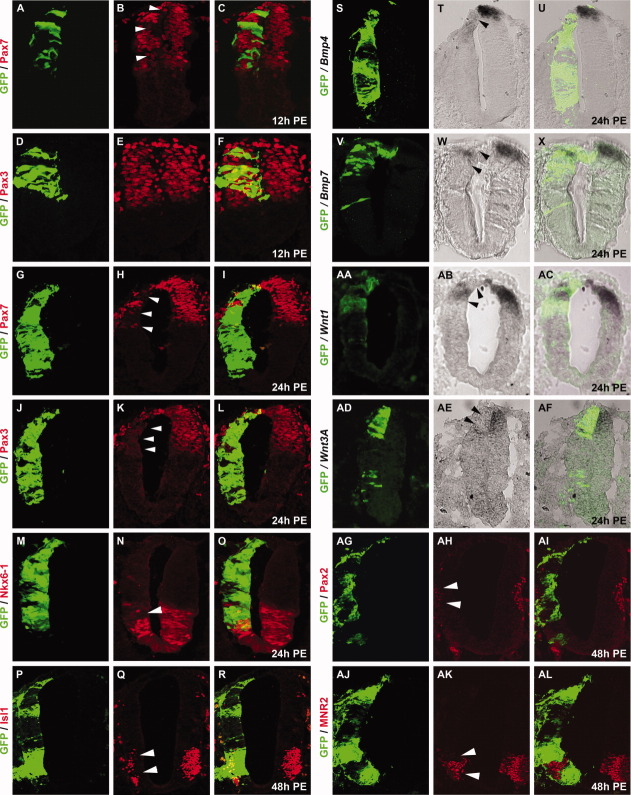
Expression of Hoxb1 represses the expression of DV markers. Detection of Pax3 ([**D–F**], [**J–L**]), Pax7 ([**A–C**], [**G–I**]), Nkx6-1 (**M–O**), Isl1 (**P–R**), Pax2 (**AG–AI**), and MNR2 (**AJ–AL**) by immunofluorescence as well as detection of *Bmp4* (**M–O**), *Bmp7* (**P–R**), *Wnt1* (**S–U**), and *Wnt3A* (**V–X**) expression by in situ hybridization on transverse sections of neural tubes 12h PE (**A–F**), 24h PE ([**G–O**], [**S–AF**]), and 48h PE ([**P–R**], [**AG–AL**]). Expression of Hoxb1 represses expression of the dorsal markers Pax7 (arrowheads in [**B**], [**H**]) and Pax3 (arrowheads in [**K**]) at 12 (**A–C**) and 24h PE (**G–I**), respectively. Consistent with a cell fate switch cells electroporated with Hoxb1 cease to express the dorsal signaling molecules *Bmp4* (arrowhead in [**T**]), *Bmp7* (arrowheads in [**W**]), *Wnt1* (arrowheads in [**AB**]), and *Wnt3A* (arrowheads in AE). Expression of Hoxb1 also represses expression of the medial marker Pax2 ([**AG–AI**], arrowheads in [**AH**]) and the ventral markers Nkx6-1 ([**M–O**], arrowhead in [**N**]), Isl1 ([**P–R**], arrowheads in [**Q**]) and NMR2 ([**AJ–AL**], arrowheads in [**AK**]) at 24h PE (**M–O**) and 48h PE ([**P–R**], [**AG–AL**]). Abbreviations: GFP, green fluorescent protein; PE, postelectroporation.

The reduction of β-III-tubulin expression and the ectopic generation of migratory NC cells suggested a switch from spinal cord to NC cell fate. To address this, we examined whether Hoxb1 expression altered the establishment of neural progenitor domains in the dorsal neural tube. The Pax3 and Pax7 transcription factors are expressed in premigratory NC cells but also in progenitors that give rise to dorsal interneurons [44, 45]. We found that Hoxb1 repressed expression of both Pax3 and Pax7 in a cell autonomous manner with early downregulation of Pax7 starting at 12h PE which continued at 24h PE (Fig. 3A–3C, 3G–3I); downregulation of Pax3 started at 24h PE (Fig. 3D–3F, 3J–3L). This finding and the Hoxb1 induced downregulation of β-III-tubulin 24 hours later (Fig. 1M–1O) suggested that a neuronal to NC cell fate switch had taken place.

Wnt and Bmp signaling have been implicated in the induction of NC fates [46–52]. Therefore, we examined whether Hoxb1 driven NC cell induction resulted from an upregulation of dorsal Wnt and/or Bmp signals. Coelectroporation of Hoxb1 with either a Tcf luciferase reporter plasmid or a Bmp luciferase reporter plasmid [29] and assay of the luciferase activity 12h PE showed that Hoxb1 did not potentiate either Wnt or Bmp signaling (data not shown). On the other hand, we found that expression of the dorsal signaling molecules *Wnt1*, *Wnt3A*, *Bmp4*, and *Bmp7* were repressed 24h PE, consistent with a cell fate switch (Fig. 3M–3X). This suggested that Hoxb1 induced NC cell fate without enhancing expression of dorsal morphogens.

We then examined whether Hoxb1 had a similar effect in medial and ventral markers. The Pax2 transcription factor establishes the identity of dorsomedial GABAergic interneurons [53], and its expression was also repressed upon Hoxb1 expression 48h PE (Fig. 3AG–3AI). Expression of the ventral neural progenitor marker Nkx6-1 [54] (Fig. 3M–3O) 24h PE and the motor neuron markers MNR2 and Isl1 [54] (Fig. 3AG–3AL) 48h PE was also repressed. These results were consistent with the observed delamination of cells from medial and ventral levels and concomitant reduced neuronal differentiation.

To examine whether Hoxb1 induces established mediators of trunk NC cell induction, survival, and delamination, we examined the expression of *Sox9*, *FoxD3*, and *Sox10* at 12, 24, and 48h PE. *Sox9* is expressed in premigratory NC cell where it promotes cell survival and together with *FoxD3* and *Snail2* induces trunk NC cells [19]. Electroporation of Hoxb1 resulted in downregulation of Sox9 by 12h PE and downregulation of *FoxD3* by 24h PE. We found no changes in *Sox10* expression at 24h and 48h PE (supporting information Fig. 4 and data not shown).

These data showed that a spinal cord to NC cell fate switch initiated by the expression of Hoxb1 in the trunk neural tube. On the other hand, Hoxb1 did not induce *Sox9* and *Foxd3* expression suggesting that it can bypass aspects of the trunk NC genetic cascade.

### Hoxb1^+^ NC Cells Are Migratory with a Broad Developmental Potential

**Figure 4:**
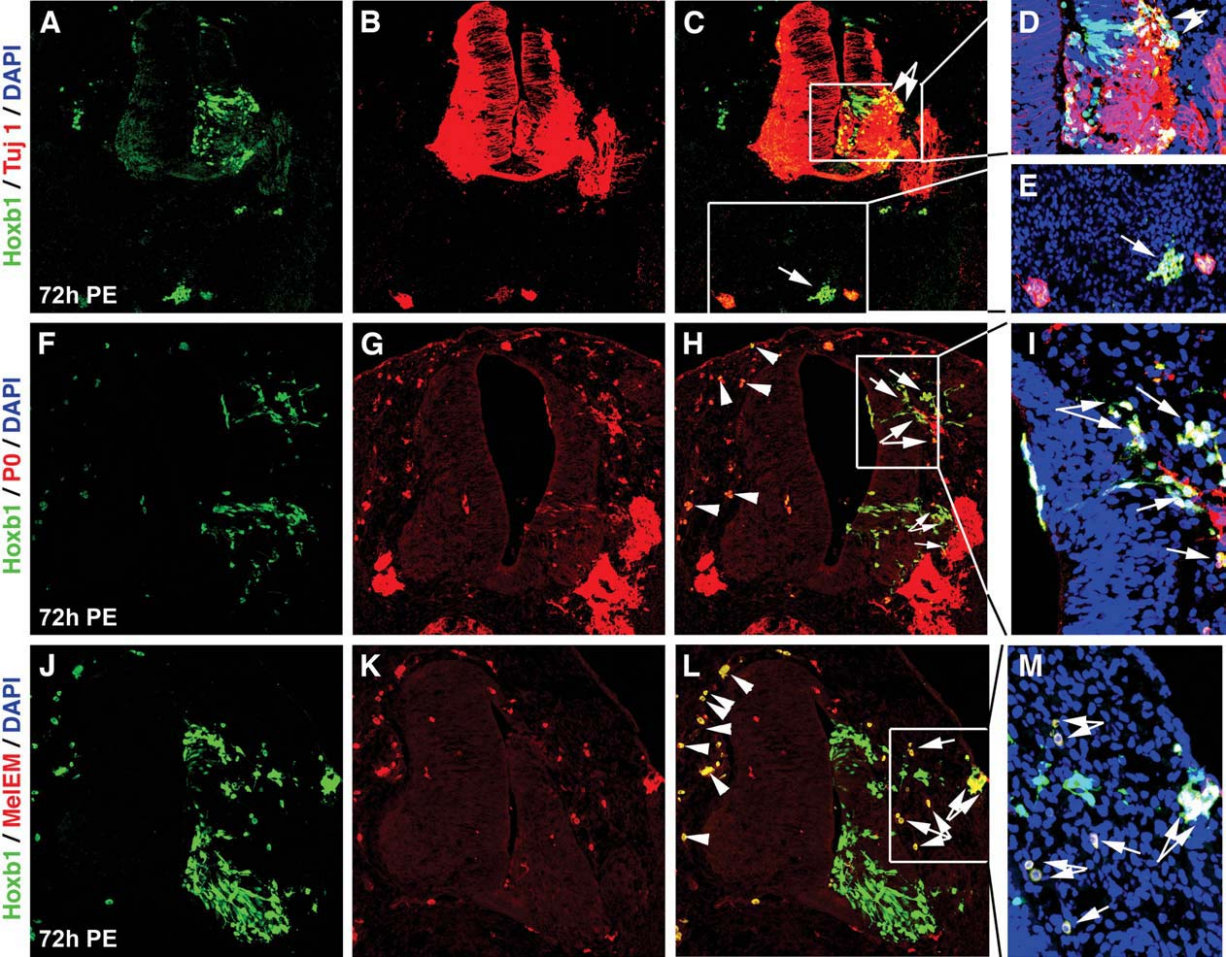
Hoxb1^+^ cells generated neuronal cells, glia progenitors, and melanocytes after delamination. Immunofluorescent detection of Hoxb1 (**A–M**), Tuj1 (**A–E**), P0 (**F–I**), and MelEM (**J–M**) on transverse sections of neural tubes 72h PE of Hoxb1. Boxed areas in (**C**), (**H**), (**L**) are shown in (**D, E**), (**I**), and (**M**), respectively, in higher magnification. Hoxb1^+^ cells generated neuronal cells (arrows in [**C–E**]), glial progenitors (arrowheads in [**H, I**]), and melanocytes (arrowheads in [**L, M**]). Tuj1^+^ and P0^+^ cells were seen also delaminating inside the lumen of the neural tube (boxed areas in [**C, H**]), whereas Hoxb1^+^/P0^+^ and Hoxb1^+^/MelEm^+^ cells migrated contralaterally as well. Abbreviations: DAPI, 4′,6-diamidino-2-phenylindole; PE, postelectroporation.

To examine the developmental potential of Hoxb1^+^ induced NC cells we examined β-III-tubulin, P0 and MelEM expression that mark the neuronal, Schwann and melanocyte lineages respectively [55–58]. Numerous Tuj1^+^/Hoxb1^+^ cells were found in the periphery 72h PE. In most cases they stayed in close proximity to the neural tube but were also found as far as the sympathetic ganglia where they occasionally formed an ectopic ganglion (Fig. 4A–4E). Hoxb1^+^/P0^+^ cells migrated into the periphery and were observed both ipsilaterally and contralaterally (Fig. 4F–4I). Hoxb1 and MelEM immunofluorescence revealed the presence of Hoxb1^+^/MelEM^+^ cells in both ipsilateral and contralateral positions (Fig. 4J–4M).

These results established that Hoxb1^+^ cells migrated away form the neural tube in ectopic positions and had a broad developmental potential. These observations are consistent with the notion that Hoxb1^+^ cells are *bona fide* NC cells.

### Bmp but Not Wnt Signaling Is Required for Hoxb1-Dependent NC Cell Induction

The downregulation of dorsal signaling molecules observed upon Hoxb1 induction was consistent with a cell fate switch but it did not address the possibility that these signals were required for Hoxb1 driven NC induction. To address this we co-electroporated Hoxb1 with constructs that blocked signal transduction of these pathways. To block canonical Wnt signaling, we co-electroporated a dominant negative *ΔN-TCF4* construct, lacking the β-catenin interaction domain [26]. Tcfs lacking this interaction domain assemble alternative complexes with transcriptional co-repressors, which act as multimeric dominant transcriptional repressors [59, 60]. Accordingly, *ΔN-TCF4* downregulates Msx1/2 at the dorsal most part of the neural tube can downregulate expression of Msx1/2 (supporting information Fig. 5). Co-electroporation of *ΔN-TCF4* with Hoxb1 did not block any of the hallmarks of Hoxb1-driven NC cell formation (supporting information Fig. 5): Snail2 and HNK-1 were still upregulated at 24 hours PE, Pax3 and Pax7 were repressed, the electroporated side was thinner and EMT occurred by 48 hours PE (supporting information Fig. 5 and data not shown).

**Figure 5:**
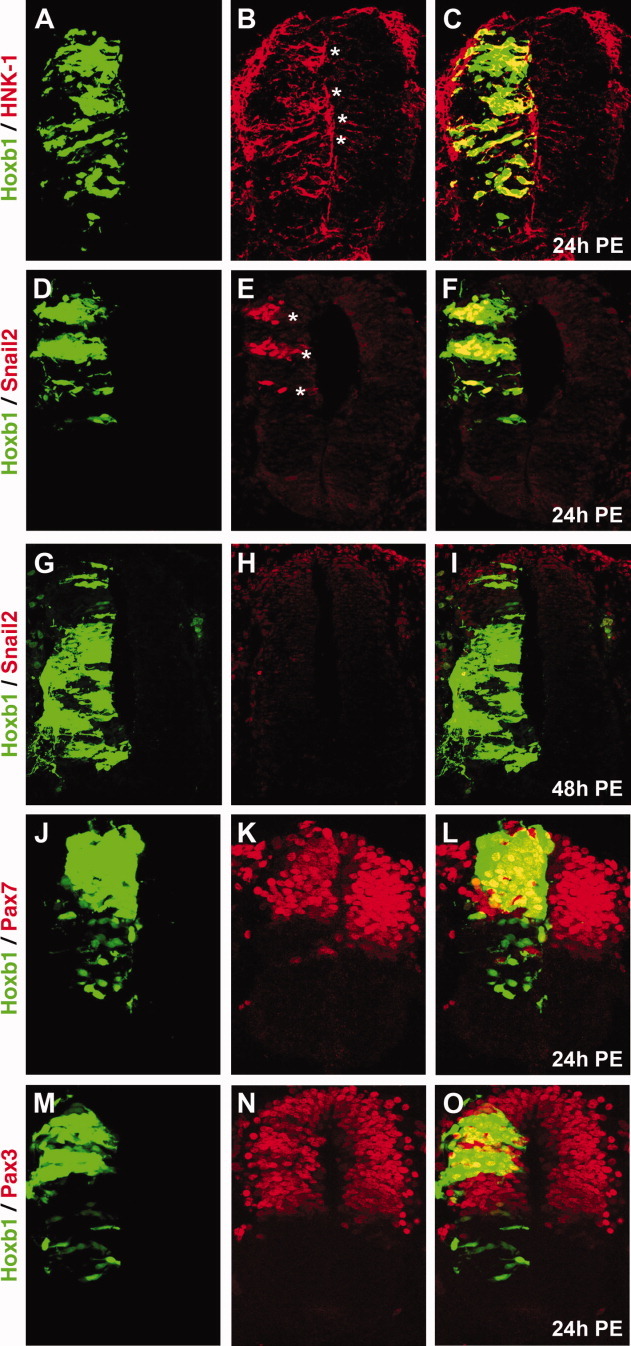
Bmp signaling was not required for Hoxb1-induced HNK-1 and Snail2 upregulation but was required for maintenance of Snail2 upregulation and repression of dorsal cell fates. Immunofluorescent detection of HNK-1 (**A–C**), Snail2 (**D–I**), Pax7 (**J–L**), and Pax3 (**M–O**) on transverse sections of neural tubes 24 ([**A–F**], [**J–O**]) and 48h PE (**G–I**) with Hoxb1 and noggin. Hoxb1 induced expression of HNK-1 ([**A–C**], asterisks in [**B**]) and Snail2 ([**D–F**], asterisks in [**E**]) in the presence of noggin at 24h PE but failed to maintain Snail2 expression at 48h PE (**G–I**). Hoxb1 no longer repressed Pax7 (**J–L**) and Pax3 (**M–O**) in the presence of noggin. Abbreviations: HNK-1, human natural killer-1; PE, postelectroporation.

To block Bmp signaling, we used a noggin expression construct [27] that alone was sufficient to completely block Bmp signaling, as assayed by luciferase assays measuring the activity of a Bmp Response Element [29] at 24 hours PE (data not shown). Hoxb1 was still able to induce Snail2 and HNK-1 expression at 24h PE in the presence of noggin (Fig. 5A–5F), but HNK-1 upregulation was noticeably weaker (compare Fig. 5A–5C with Fig. 1D–1F). Furthermore, Snail2 upregulation was not maintained past 24h PE (Fig. 5G–5I) suggesting that the maintenance of Hoxb1-mediated Snail2 upregulation was Bmp dependent. Consequently, EMT was blocked in Hoxb1/noggin coelectroporated embryos and the neural tube retained its normal size in the electroporated side (data not shown). Moreover, the cell fate switch induced by Hoxb1 was essentially blocked in the presence of noggin (Fig. 5J–5O). On the other hand, constitutive activation of Bmp signaling using CA BMPR IA [61] to coelectroporate with Hoxb1 did not affect Hoxb1-induced NC formation and delamination (data not shown).

These results suggested that Wnt signaling was not required for Hoxb1-mediated NC induction, whereas Bmp signaling was required for strong HNK-1 induction, maintenance of the Snail2 upregulation, and EMT, as well as Hoxb1-mediated dorsal cell fate switch.

### Hoxb1 Interacts with Notch Signaling to Induce NC

**Figure 6:**
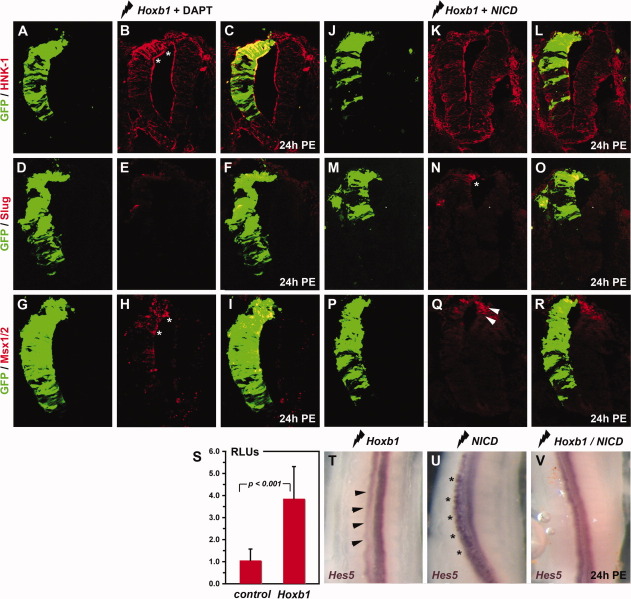
Optimal levels of Notch signaling were required for Hoxb1-mediated induction of neural crest cell fates and Hoxb1 itself modulates Notch signaling. Immunofluorescent detection of HNK-1 ([**A–C**], [**J–L**]), Snail2 ([**D–F**], [**M–O**]), and Msx1/2 ([**G–I**], [**P–R**]) on transverse sections of neural tubes, luciferase assays for CSL transcriptional activity (**S**) and *Hes5* detection by in situ hybridization (**T–V**). All assays were done 24h PE of Hoxb1 in the presence of DAPT (**A–I**), of Hoxb1 with NICD ([**J–R**], [**V**]), of Hoxb1 alone (**S**, **T**), or NICD alone (**U**). Hoxb1 in the presence of DAPT-activated HNK-1 only at dorsal levels ([**A–C**], asterisks in [**B**]), failed to activate Snail2 (**D–F**) but reactivated Msx1/2 at dorsal levels ([**G–I**], asterisks in [**H**]). Hoxb1 in the presence of NICD failed to activate HNK-1 (**J–L**), reactivated Snail2 only marginally at dorsal levels ([**M–O**], asterisk in [**N**]) and repressed Msx1/2 ([**P–R**], arrowheads in [**Q**]). Hoxb1 electroporation increased CSL transcriptional activity (**S**) (*p* < .001). Hoxb1 on its own repressed *Hes5* (arrowheads in [**T**]) in contrast to NICD that activated it (asterisks in [**U**]). Coelectroporation of Hoxb1 and NICD restored normal levels of *Hes5* expression (**V**). Abbreviations: DAPT, *N*-[*N*-(3,5-difluorophenacetyl-L-alanyl)]-S-phenylglycine τ-butyl ester; GFP, green fluorescent protein; HNK-1, human natural killer-1; NICD, Notch intracellular domain; PE, postelectroporation; RLUs, relative light units.

Active Notch signaling has been associated with maintenance of the neural progenitor state [62, 63] and with induction of NC [64, 65]. Treatment of HH10-11 chick embryos with the γ-secretase inhibitor DAPT at 50 μM completely repressed expression of Msx1/2 within 24 hours (Fig. 6G–6H and data not shown). Hoxb1 remained equally effective in repressing the expression of Pax3 and Pax7 in the presence of DAPT (data not shown) and it was able to reactivate expression of Msx1/2 mainly at dorsal levels of the neural tube in a cell autonomous manner (Fig. 6G–6I). However, in the presence of DAPT, Hoxb1-mediated induction of HNK-1 was confined to dorsal levels of the neural tube (Fig. 6A–6C), induction of Snail2 was completely abolished (Fig. 6D–6F) and the electroporated side of the neural tube retained normal thickness (data not shown). Prompted by the finding that the NICD on its own induces a small but consistent ventral shift in the expression domain of Msx1/2 (data not shown), we examined whether Hoxb1-induced NC fate commitment entailed potentiation of Notch signaling. Coelectroporation of a CSL-dependent Notch luciferase reporter [30] with Hoxb1 and assay of the luciferase activity 24h PE showed that Hoxb1 potentiated Notch signaling (Fig. 6S) apparently elevating it above a required threshold to induce NC. We then examined whether higher levels of Notch signaling could further increase Hoxb1-driven NC induction. When coelectroporated with NICD, Hoxb1 still repressed expression of both Pax3 and Pax7 and reduced neurogenesis on the electroporated side (data not shown). Surprisingly, coelectroporation of Hoxb1 and NICD strongly repressed Msx1/2 expression (Fig. 6P–6R) and failed to induce Snail2 expression (Fig. 6M–6O). Furthermore, induction of HNK-1 was nearly abolished and persisted only occasionally at dorsal levels (Fig. 6J–6L). Thus, Hoxb1-induced switch of spinal cord progenitors to NC fates required intermediate levels of Notch signaling.

Notch signaling maintains the neural progenitor state and Hoxb1-mediated commitment to NC cell fate may entail repression of selected effectors. Thus, we assayed expression of *Hes5*, a key effector gene of Notch signaling in the neuroepithelium [66] contributing to progenitor maintenance and expressed in the neural tube [67]. Electroporation of Hoxb1 repressed *Hes5* expression and that repression was released by NICD coelectroporation (Fig. 6T–6V). This observation also provides a likely mechanism for the reduced mitotic rate following *Hoxb1* electroporation.

These findings suggested that Hoxb1-mediated induction of NC fate but not cell fate switch is dependent upon optimal levels of Notch signaling. Hoxb1 itself potentiates Notch signaling raising it above a required threshold and induces cell fate commitment by repressing *Hes5*.

### Hoxa2 Has a Similar Potential to Hoxb1 to Induce NC Cell Fates

**Figure 7:**
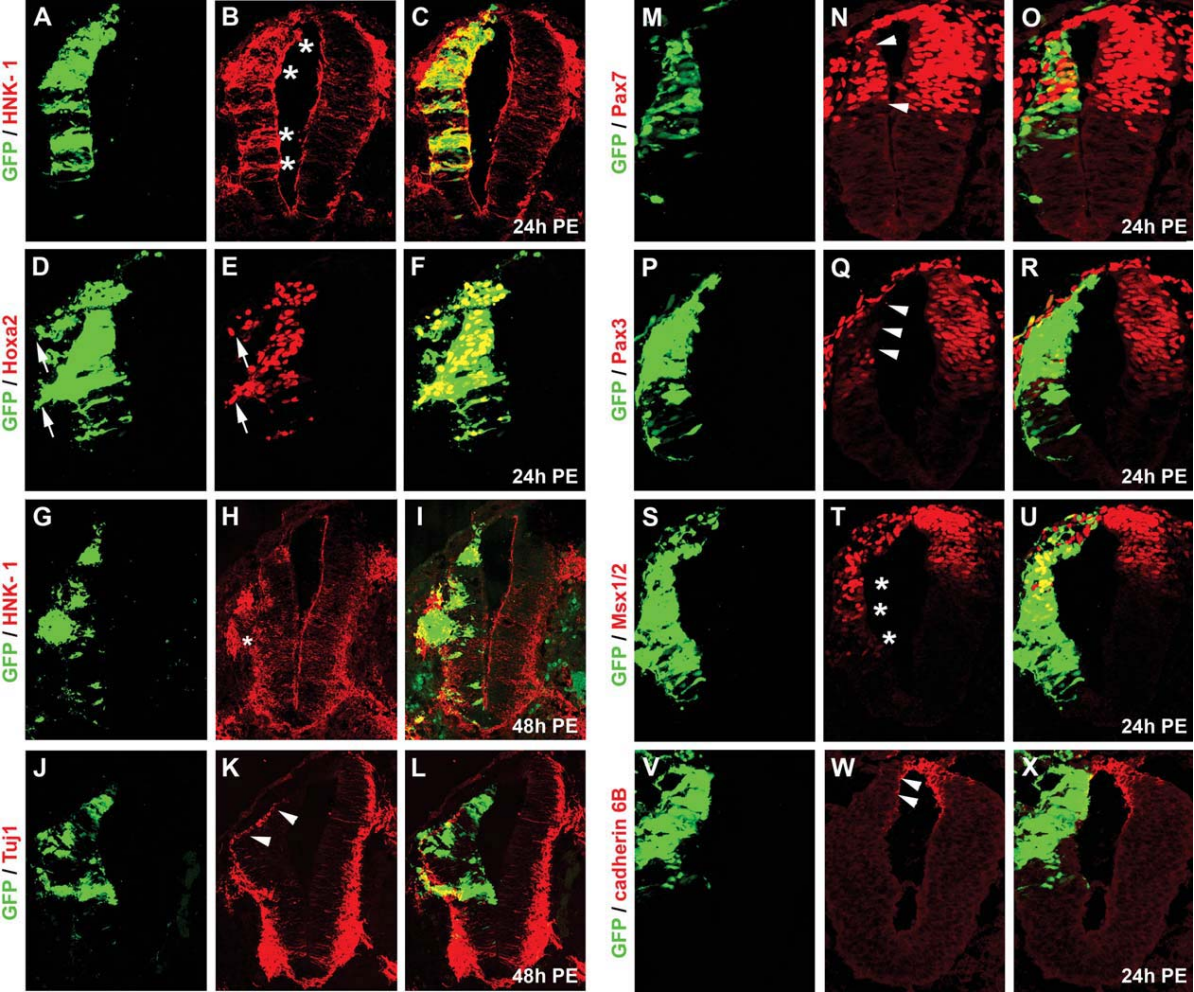
Expression of Hoxa2 in the trunk neural tube induced a neuronal progenitor to neural crest cell fate switch and limited epithelial-to-mesenchymal transition. Immunofluorescent detection of HNK-1 ([**A–C**], [**G–I**]), Hoxa2 (**D–F**), Tuj1 (**J–L**), Pax7 (**M–O**), Pax3 (**P–R**), Msx1/2 (**S–U**), and cadherin 6B (**V–X**) was performed on transverse sections of neural tubes 24h ([**A–F**], [**M–X**]) and 48h PE (**G–L**) with Hoxa2. Hoxa2 cell autonomously upregulated HNK-1 ([**A–C**] and [**G–I**], asterisks in [**B**] and [**H**]). Hoxa2^+^ cells delaminated 24h PE ([**D–F**], arrows in [**D, E**]). Hoxa2 strongly repressed neuronal differentiation 48h PE ([**J–L**], arrowheads in [**K**]). Hoxa2 cell autonomously repressed Pax7 ([**M–O**], arrowheads in [**N**]), Pax3 ([**P–R**], arrowheads in [**Q**]) and cadherin 6B ([**V–X**], arrowheads in [**W**]), whereas it upregulated Msx1/2 ([**S–U**], asterisks in [**T**]). Abbreviations: GFP, green fluorescent protein; HNK-1, human natural killer-1; PE, postelectroporation.

Hoxa2 plays a pivotal role in patterning the NC of the second branchial arch of the head [68], but a role in NC cell induction has not been documented. Hoxa2 was able to upregulate HNK-1 expression in the trunk to a similar extent as Hoxb1 24h PE (Fig. 7A–7C). Furthermore, at 24h PE Hoxa2^+^ cells were occasionally observed leaving the neural tube from both dorsal and medial positions (Fig. 7D–7F) but HNK-1 upregulation was not sustained at high levels 48h PE. Similar to Hoxb1, β-III-tubulin expression was substantially reduced 48h PE (Fig. 7G–7I) and there was a repression of both Pax7 (Fig. 7M–7O) and Pax3 (Fig. 7P–7R) expression by 24h PE establishing that a spinal cord to NC cell fate switch was taking place. In addition, in Hoxa2 electroporated embryos expression of Msx1/2 was extended to medial levels of the neural tube (Fig. 7S–7U) and the neural tube itself was thinner (data not shown). Cell adhesion properties also changed, consistent with NC cell fate (Fig. 7V–7X). Hoxa2 on its own did not induce high levels of Snail2 expression at either 24h or 48h PE (data not shown). This might explain the lower levels of EMT in the Hoxa2 electroporated embryos. Therefore, Hoxa2 has the capacity to induce NC fates but weaker capacity than Hoxb1 to induce EMT.

## DISCUSSION

Several studies have demonstrated the role of Hox genes in patterning NC cells, particularly, in the cranial region of the body [20]. Despite indications from loss-of-function studies and ES-derived NS cells that anterior *Hox* genes may participate in NC induction [21, 23] direct evidence was lacking. Here, we provide evidence that anterior *Hox* genes can mobilize the NC genetic cascade leading up to and including EMT. Among the genes examined, *Hoxb1* had the strongest potential for inducing NC cell fates by interacting with the Bmp and Notch signaling pathways. We propose that anterior *Hox* genes sensitize anterior neural cells to NC-inducing signals and participate in key aspects of the cranial NC genetic cascade.

### Differences Between Trunk and Cranial NC and the Role of Anterior *Hox* Genes

Several lines of evidence suggest differences in the NC genetic cascade in the trunk and cranial region [3]. Snail2 is necessary and sufficient for NC generation and delamination in cranial regions but not in the trunk [14, 36]. Loss of Msx1 and Msx2 resulted in reduced NC survival and NC patterning defects specifically in cranial and cardiac NC [31]. Sox9 and Sox10 play a central role early in the trunk NC genetic cascade [3, 12] but less so in the early steps of cranial NC specification and delamination [69–72]. Collectively, these studies suggested that the *Msx1/2* and *Snail* genes have a critical role in early specification and survival of cranial NC cells, whereas Sox9 and Sox10 play a correspondingly more prominent role in the trunk NC. Accordingly, Hoxb1 induced upregulation of Msx1/2 and Snail2 but not *Sox9* or *Sox10*. This is consistent with functional overlap between anterior *Hox* genes and posterior NC specifiers. In this regard, it is worth noting that both FoxD3 and Sox9 were necessary to stabilize Snail2 in ectopic expression experiments [73]. In contrast, Hoxb1-mediated induction of Snail2 occurred more rapidly and lasted longer than that observed after electroporation of Sox9 or FoxD3 [19, 37]. Snail2 is a labile protein, its stability is enhanced through the action of FoxD3 and Sox9 [73]. Our findings suggest that Hoxb1 can substitute for FoxD3 and Sox9 in this function. The Hoxb1-mediated repression of endogenous Sox9 and FoxD3 suggested that Hoxb1 plays a distinct role in promoting delamination and differentiation of premigratory NC cells in the neural tube. Notably, Sox9 is downregulated before delamination [37], whereas maintenance of FoxD3 expression in the cranial NC blocks migration and differentiation [74].

Extended functional redundancy in NC specification may have masked the suggested role of anterior *Hox* genes in this process. Single or double Hox mutants display patterning defects but not NC specification or migration defects [20]. Eliminating the Hox-binding partners *pbx* genes in zebrafish lead to a transformation of the r2-r7 territory into r1 [75]. A similar experiment in frogs whereby the entire paralogous group 1 was knocked down resulted in NC migratory but not specification defects concomitant with an expansion of the *Gbx2* expression in the *r2–r7* territory. Gbx2 has been recently identified as a NC inducer accounting for the presence of NC in the transformed territory [76, 77]. However, an r4-specific triple Hox loss-of-function mutation resulted in loss of the expression of all r4 NC-derived molecular markers and the structures derived from r4 NC populating the second arch. Reciprocal grafts between mutant and wt embryos showed that these defects were cell autonomous to the mutant r4 suggesting a role for anterior *Hox* genes in NC specification [21, 22]. Here, we show by gain-of-function experiments that anterior *Hox* genes had a similar but variable capacity in inducing NC cell fates when expressed in the trunk. Hoxb4, expressed in the postotic region of the hindbrain, next to RA generating axial mesoderm [78] failed to induce NC character. These findings further support the existence of functional specificity among *Hox* genes [79–81].

### Anterior *Hox* Genes May Sensitize Cells to NC-Inducing Signals

A gradient of Bmp activity specifies the border of the neural plate as the presumptive NC territory and subsequently a combination of Wnt, Fgf, and RA signaling transforms the border of the neural plate into NC [4, 5]. Wnt, Fgf, and RA signals have been implicated in the acquisition of caudal neural identity [82–86]. The localization of the tissues expressing these signals [87], the expression patterns of RA degrading enzymes [88, 89], and the expression patterns of Wnt signaling antagonists [90] generate a gradient of posteriorizing, and therefore, NC-inducing activities that leave the hindbrain region at a relative deficit of these signals [91]. We hypothesized that Hox homeobox genes expressed in the anterior neural plate compensate by sensitizing cells to NC-inducing signals. Consistent with this, Noggin blocked the Hoxb1 NC-inducing capacity, at least partly by abolishing Hoxb1-mediated Snail2 stabilization, suggesting an important input of Bmp signaling in the late steps of the NC genetic cascade.

On the other hand, blocking Notch signaling with DAPT or constitutively activating Notch signaling with NICD had a similar effect suggesting that intermediate levels of Notch signaling are necessary for Hoxb1-mediated NC specification and EMT. We propose that Hoxb1 may promote NC specification by elevating Notch signaling above a possible threshold required for NC induction. In parallel, it may modify the Notch signaling functional readout by selectively repressing or activating some of its effectors. The observed repression of *Hes5* may disrupt the oscillation of Notch activity that maintains neuronal progenitors [63] forcing early cell fate commitment. The observed reduction of proliferation is consistent with early cell fate commitment, which combined with the Bmp-dependent repression of neuronal progenitor fates, may propel neuroepithelial cells toward NC fate.

### EMT in Development and Cancer and the Implication of *Hox* Genes

Many of the factors that participate in the NC genetic cascade, are also involved in tumor progression and cancer cells share many characteristics with NC cells, particularly, regarding EMT. Premigratory NC cells and nonmetastatic tumor cells form an epithelium with a typical apical-basal polarity loss of which is a prerequisite for migration initiated with the loss of tight junctions located in the apical zone [92]. Occludin and claudins are components of tight junctions and their expression progressively decreases both in NC and tumor cells [93]. Interestingly, occludin and claudin 12 were repressed in ESC-derived neuroepithelial cells in response to Hoxb1 induction (Gouti and Gavalas, unpublished data). At the onset of tumor metastasis and NC migration and concurrently with loss of cell polarity the profile of cadherin-expression changes. Consistent with inducing EMT electroporation of Hoxb1 results in loss of cell polarity illustrated by the loss of cadherin 6b and N-cadherin expression and upregulation of cadherin 7. Changes in N-cadherin and cadherin 7 expression are stronger in the dorsal half of the neural tube possibly reflecting the dependence of Hoxb1-induced NC and EMT on dorsal Bmp signals. Altered *Hox* gene expression has been associated with tumor progression [94]. HOXB7 and HOXB13 have been linked to increased instances of metastasis in breast and ovarian cancers and their overexpression in cell lines enhances many features of EMT [95, 96]. Here, we provided evidence that *Hox* genes can directly induce EMT in vivo in concert with Notch and Bmp signaling that have also been implicated in tumor progression. Thus, we propose that some *Hox* genes may be important players in EMT during tumor progression.

## CONCLUSION

Several studies have demonstrated the role of Hox genes in patterning NC cells, particularly in the cranial region of the body, but despite indications from loss-of-function studies that anterior Hox genes may participate in NC induction, direct evidence was missing. Dorsalization of ES-derived NS by dorsal morphogenetic signals resulted in strong upregulation of the key NC-inducing gene *Msx1* selectively in Hoxb1^+^ but not Hoxb1^−^ NS cells, suggesting that Hox genes may indeed participate in NC induction. We addressed this hypothesis by expressing Hoxb1 and other anterior Hox genes in the caudal neural tube of the developing chick embryo. We found that anterior Hox genes play a central role in NC cell specification by rapidly inducing the key transcription factors Snail2 and Msx1/2 and mobilizing the complete NC specification genetic program. Hoxb1-induced NC specification and EMT depended on BMP signaling and optimal levels of Notch signaling. Hoxa2 had a similar capacity in inducing NC cell character but a weaker one in inducing EMT. Other anterior Hox genes, such as *Hoxa1* and *Hoxb2*, but not the posterior gene *Hoxb4*, also had the capacity to induce NC cell character. Thus, deregulated expression of certain Hox genes in combination with specific signaling pathways may be a key event in inducing EMT during tumor progression.

## Supplementary material

Supplementary Figure 1. Expression of Hoxb1 in the trunk region of the neural tube reduces mitotic rates and results in thinning of the neural tube. Immunohistochemical detection of BrdU incorporation on transverse sections of the neural tube 6h PE of Hoxb1 (A-C) and quantitation shows reduced incorporation in Hoxb1+ cells as compared to control cells (F) (p<0.001) and results in thinner neural tube on the electroporated side apparent by 24h PE (compare brackets in D, E).

Supplementary Figure 2. Expression of anterior Hox genes in the trunk neural tube may induce upregulation of HNK-1. Immunohistochemical detection of HNK-1 (A-I) was performed on transverse sections of neural tubes 24h PE with Hoxa1 (A-C), Hoxb2 (D-F) and Hoxb4 (G-I). Expression of Hoxa1 and Hoxb2 upregulated HNK-1 in a cell autonomous manner (A-F, asterisks in B and E) but electroporation of Hoxb4 had no effect on HNK-1 expression (G-I).

Supplementary Figure 3. Expression of Hoxb1 in the midbrain region induces a neuronal to neural crest switch. Immunohistochemical detection of HNK-1 (A-C), Snail2 (D-F), Msx1/2 (G-I) and Tuj1 (J-L) was performed on transverse sections of the midbrain 24h PE with Hoxb1. Expression of Hoxb1 upregulated HNK-1 (A-C, asterisks in B), Snail2 (D-F, asterisks in E) and Msx1/2 (G-I, asterisks in H) whereas it repressed expression of the neuronal marker Tuj1 (J-L, arrowheads in K).

Supplementary Figure 4. Expression of Hoxb1 in the trunk region represses expression of Sox9 and FoxD3. Detection of Sox9 (A-C) and FoxD3 (D-F) expression by in situ hybridisation on transverse sections of neural tubes 12h PE (A-C) and 24h PE (D-F). Ectopic expression of Hoxb1 represses expression of Sox9 12h PE (arrowheads in B) and FoxD3 24h PE (arrowhead in E).

Supplementary Figure 5. Blocking Wnt signalling does not affect the ability of Hoxb1 to induce NC cell fates. Immunohistochemical detection of Msx1/2 (A-C), HNK-1 (D-F), Snail2 (G-I), Pax7 (J-L) and Pax3 (M-O) on transverse sections of neural tubes 24h PE (A-O) of dnTCF4 alone (A-C) or in combination with Hoxb1 (D-O). Electroporation of dnTCF4 alone repressed Msx1/2 expression on the electroporated side (A-C, arrowheads in B). However, ectopic expression of Hoxb1 in the presence of dnTCF4 still resulted in ectopic expression of HNK-1 (D-F, asterisks in E), ectopic expression of Snail2 (G-I, asterisks in H) and repression of Pax7 (J-L, arrowheads in K) and Pax3 (M-O, arrowheads in N).
