# Supplementary figures and images for: Anterior Hox Genes Interact with Components of the Neural Crest Specification Network to Induce Neural Crest Fates
Source: Stem Cells. 2011 Mar 23;29(5):858–70. doi: 10.1002/stem.630 (PMC3184476; doi:10.1002/stem.630)

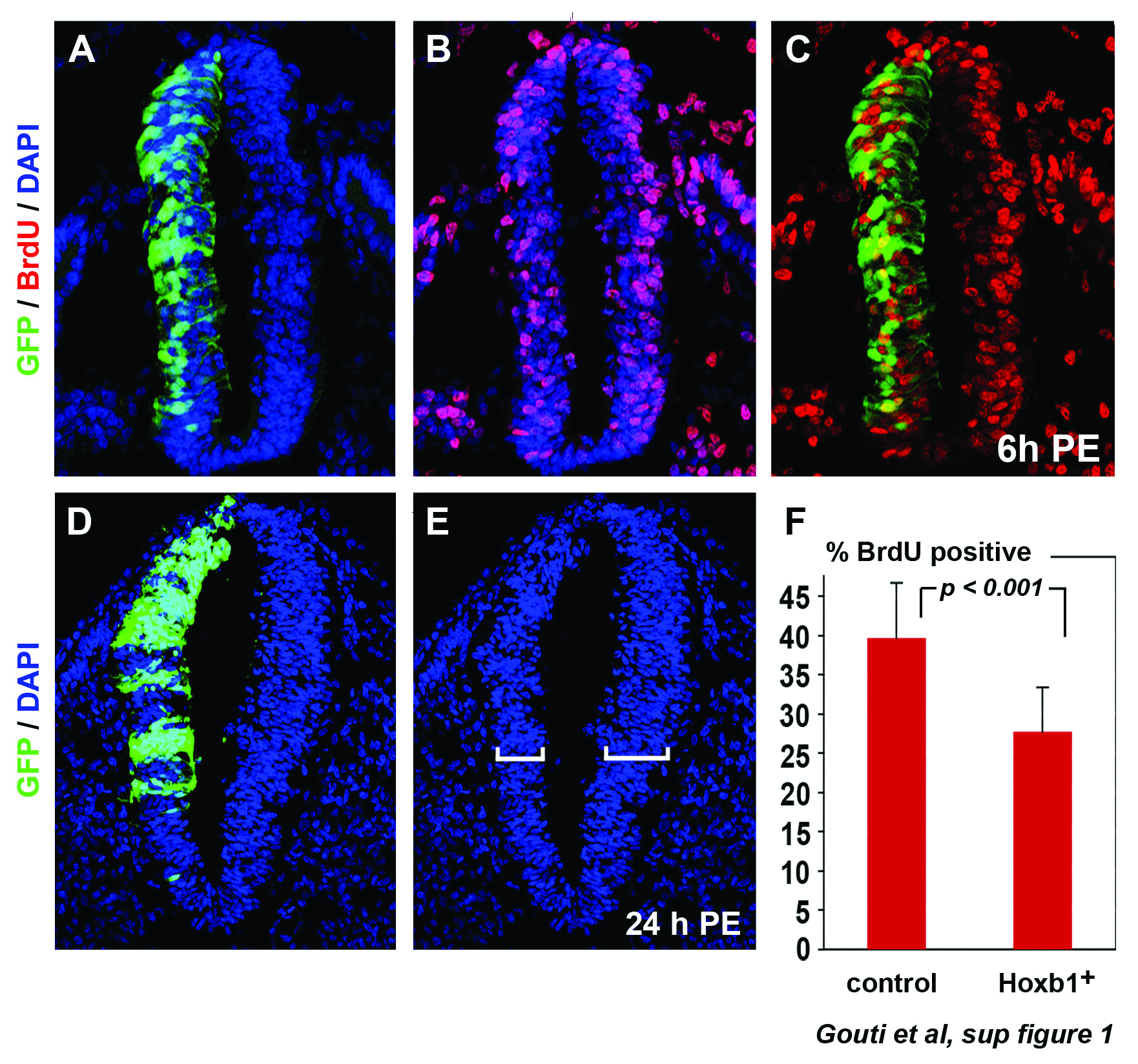

Supplement: Supplementary file 1 [file stem0029-0858-SD1.tif]

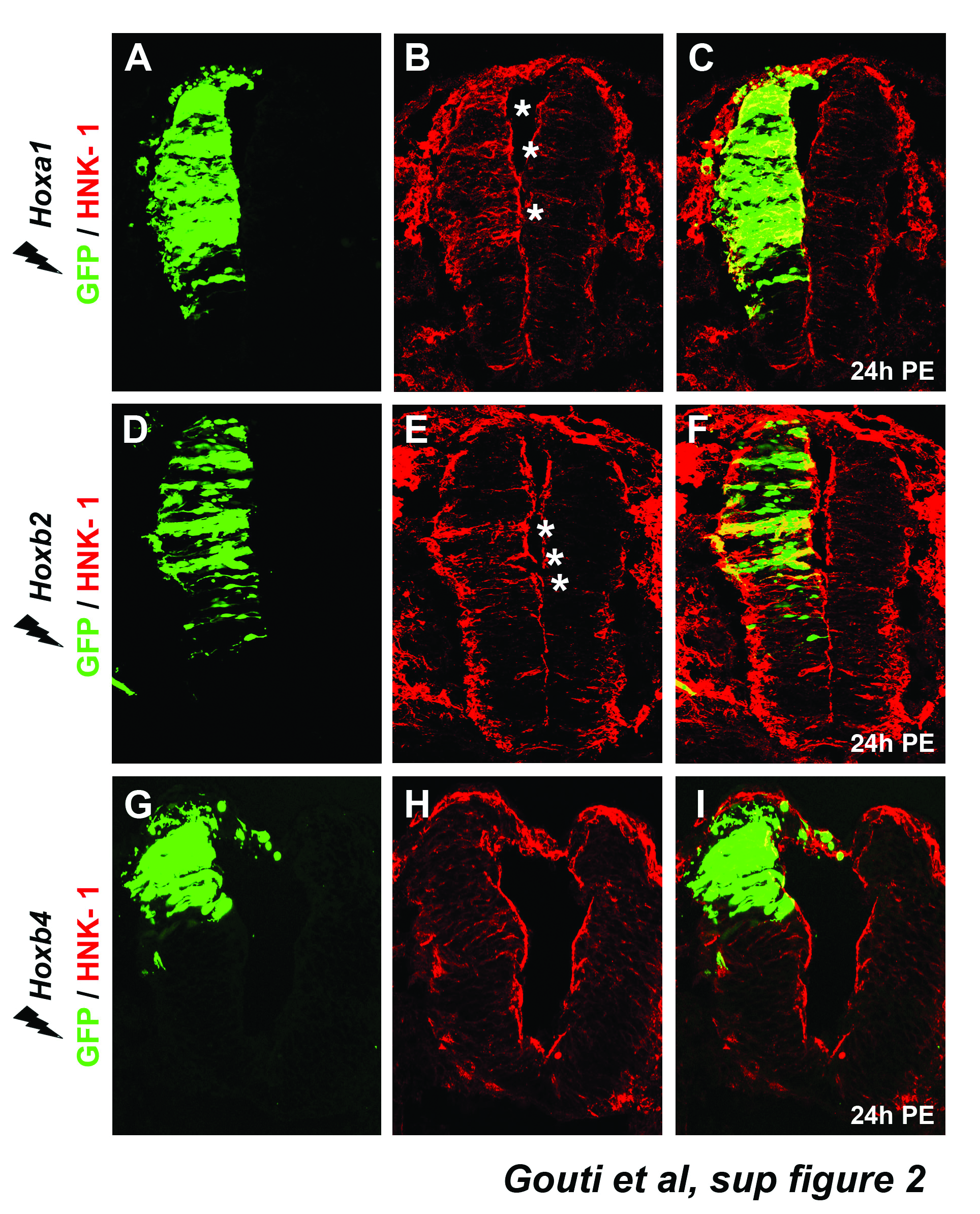

Supplement: Supplementary file 2 [file stem0029-0858-SD2.tif]

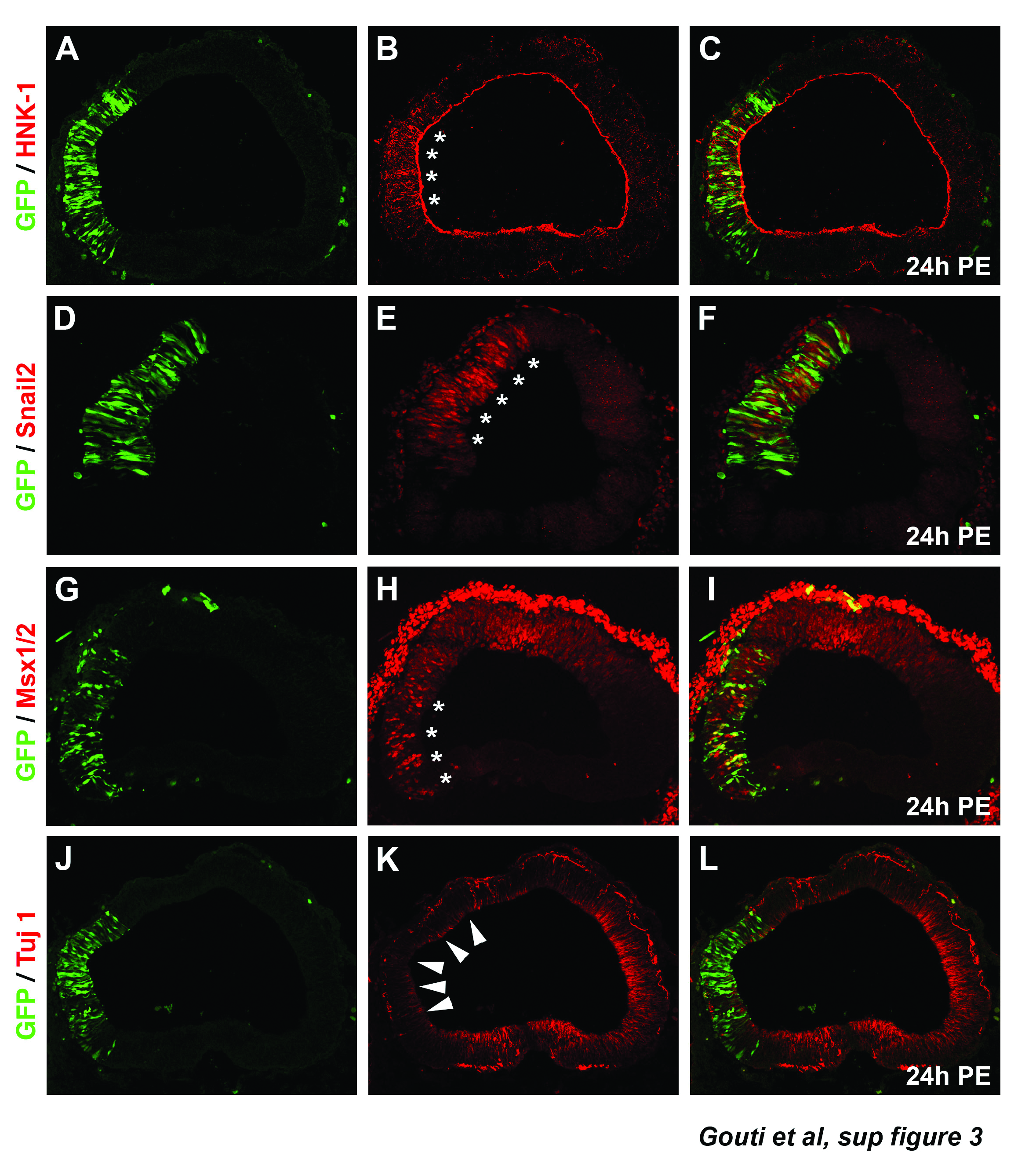

Supplement: Supplementary file 3 [file stem0029-0858-SD3.tif]

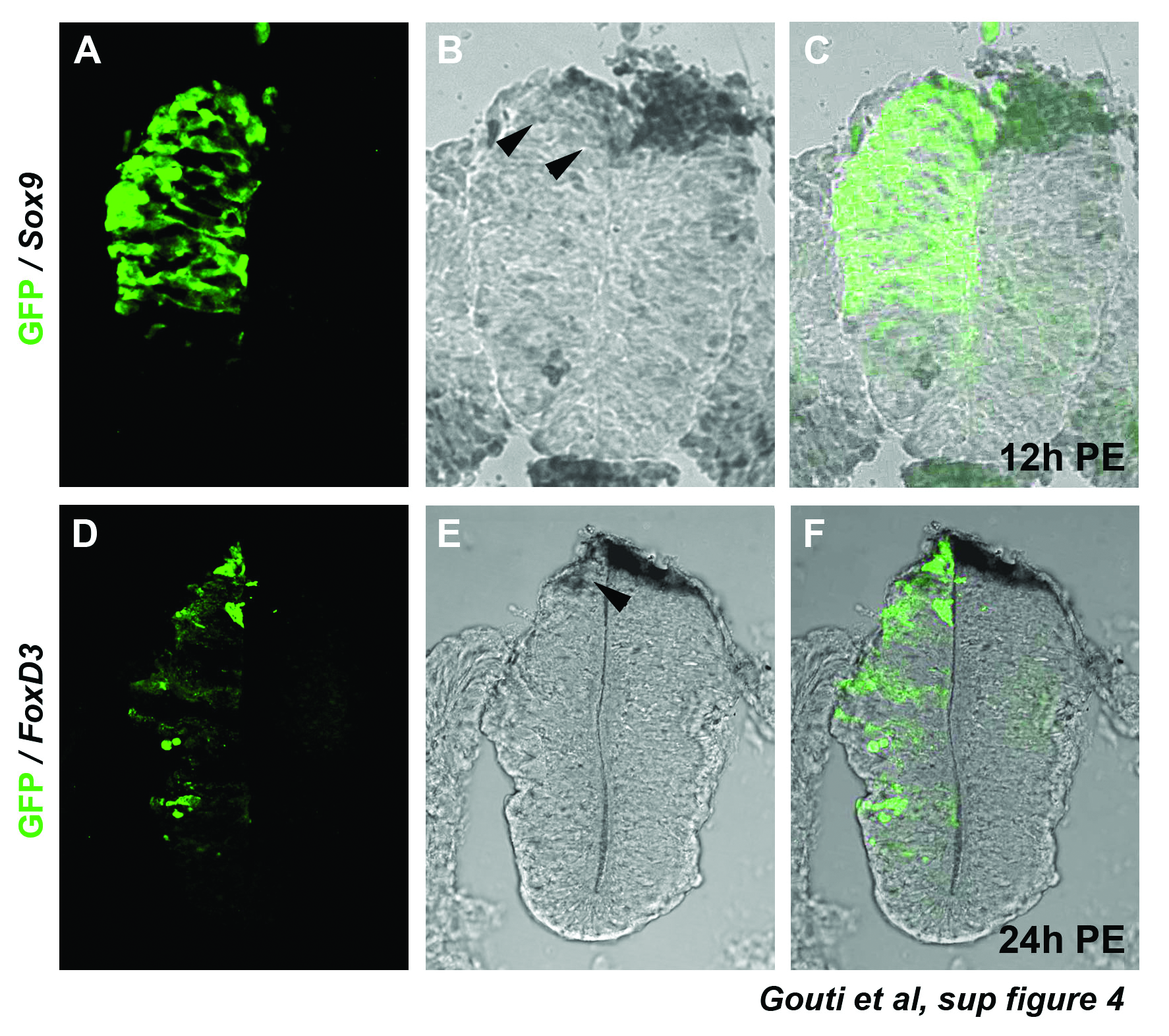

Supplement: Supplementary file 4 [file stem0029-0858-SD4.tif]

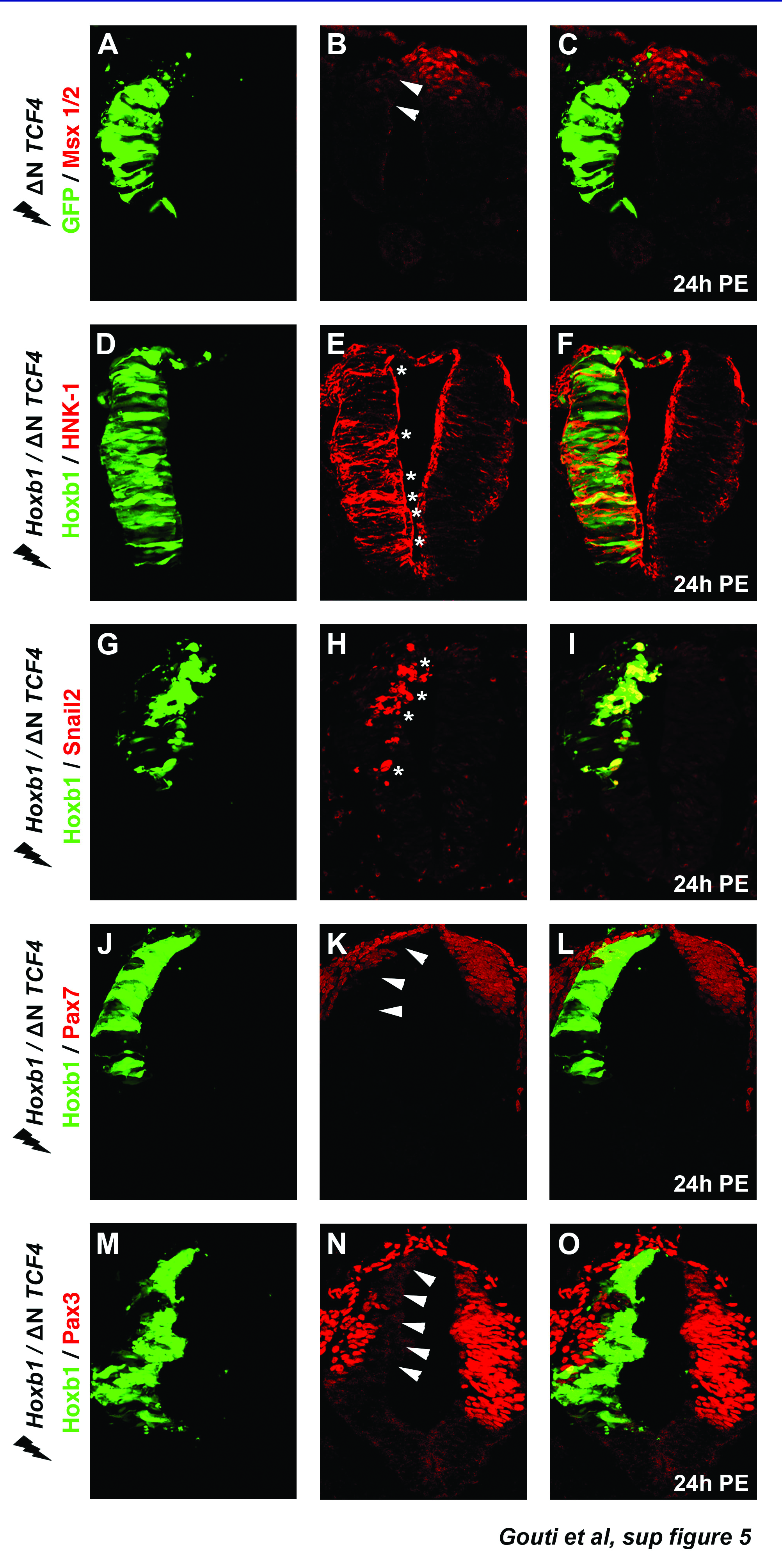

Supplement: Supplementary file 5 [file stem0029-0858-SD5.tif]
