# Supplementary material for: Anterior Hox Genes Interact with Components of the Neural Crest Specification Network to Induce Neural Crest Fates
Source: Stem Cells. 2011 Mar 23;29(5):858–70. doi: 10.1002/stem.630 (PMC3184476; doi:10.1002/stem.630)
Supplement: Supplementary file 6 [file stem0029-0858-SD6.doc]

**Supplementary materials and methods**

Chick in situ hybridization probes were *Sox9*, *Sox10*, *FoxD3*, *Bpm4* and *Bmp7* ^1^, *Wnt1* and *Wnt3a* (kind gift from N. Itasaki), Hoxa2 ^2^and *Hes5* ^3^.

Primary antibodies used were: rabbit anti-Hoxb1, 1:400 (Covance), mouse anti-HNK-1, 1:1000 (Becton-Dikinson), mouse anti-Tuj1, 1:1000 (Covance), mouse anti-HA, 1:200 (Cell Signaling), mouse anti-Cleaved Caspase-3, 1:500 (Cell Signaling) and from Developmental Studies Hybridoma Bank (DSHB) mouse anti-MelEM, 1:100 mouse anti-P0, 1:100, mouse anti-Snail2, 1:200, mouse anti-Msx1/2, 1:200, mouse anti-cadherin 6, 1:100, mouse anti-cadherin 7b, 1:100, mouse anti-N-cadherin, 1:200, mouse anti-Pax7, 1:50, mouse anti-Pax3, 1:250 and mouse anti-Hoxb4, 1:100. Secondary antibodies were anti-mouse and anti-rabbit Alexa 488 or Alexa 568 or Alexa 633 conjugated goat antibodies (Molecular Probes) used at 1:500.

**References**

1. Cheung M, Briscoe J. Neural crest development is regulated by the transcription factor Sox9. Development*.* 2003;130:5681-5693.

2. Kutejova E, Engist B, Self M, et al. Six2 functions redundantly immediately downstream of Hoxa2. Development*.* 2008;135:1463-1470.

3. Politis PK, Makri G, Thomaidou D, et al. BM88/CEND1 coordinates cell cycle exit and differentiation of neuronal precursors. Proc Natl Acad Sci U S A*.* 2007;104:17861-17866.
